# Influence of the Knee and Hip Joint Angles on the Knee Extensor Muscles and Tendon Shear Wave Velocity in Men and Prepubertal Boys

**DOI:** 10.1155/tsm2/5557335

**Published:** 2025-12-23

**Authors:** Baptiste Chanel, Carole Cometti, Nicolas Babault

**Affiliations:** ^1^ Centre d’Expertise de la Performance, Faculté des Sciences du Sport, Université Bourgogne Europe, Dijon, F-21000, France; ^2^ INSERM, CAPS UMR 1093, Université Bourgogne Europe, Dijon, 21000, France, inserm.fr

**Keywords:** children, quadriceps, stiffness, ultrasonography

## Abstract

**Introduction:**

This study aimed to compare the influence of the knee and hip joint angles on the quadriceps muscles and tendon shear wave velocity (SWV) between men and prepubertal boys.

**Methods:**

Ten prepubertal boys and ten men participated in one experimental session, which included SWV measurements at rest for the vastus lateralis (VL), the rectus femoris (RF), and the patellar tendon (PT). Each volunteer was tested in ten randomized positions, including five knee joint angles (30, 50, 70, 90, and 110°, 0° = full extension) and two hip joint angles (0° and 80°, 0° = full extension). The SWV values were analyzed independently with a three‐way ANOVA, while considering the knee angle, hip angle, and the age group.

**Results:**

Significant knee angle × age interactions were observed for VL (*p* = 0.008), RF (*p* = 0.008), and PT (*p* < 0.001). Boys and men did not present a difference in VL and RF SWV. However, boys exhibited significantly lower PT SWV values than men from a 30° to 90° knee flexion angle (*p* < 0.001). Additionally, a hip angle × knee angle interaction (*p* < 0.001) has been observed for RF SWV and highlighted greater values with a 0° hip joint angle, whatever the knee joint angles.

**Conclusion:**

These results suggested that boys and men present similar muscle elastic properties in VL and RF. However, lower SWV was observed for PT in boys up to 90° knee joint flexion.

## 1. Introduction

Shear wave elastography (SWE) is a technique characterizing the myotendinous elastic properties [1]. It is used in clinical settings to identify diseases such as tendinopathies [2, 3]. SWE is also used to identify chronic adaptations induced by training or short‐term alterations associated with a fatiguing exercise [4]. The main concept of SWE is to induce an acoustic radiation impulse over a tissue and to quantify the resultant velocity of the shear waves. This variable is called shear wave velocity (SWV). SWE presents the asset to permit the distinction of most myotendinous structures, as well as the evaluation of local adaptation.

In contrast to the fast emergence of SWE in adult populations, the literature is scarce in prepubertal children [5]. However, SWE could be useful for exploring children’s myotendinous system and revealing growth‐related changes at rest or during voluntary contractions. With children, SWE was mostly used in clinical settings [6–8], to characterize pathological tissue [9] and establish standard values [10]. With healthy children, no age influence was observed from 2 to 12.6 years old lateral gastrocnemius SWV [11]. This result was confirmed for the rectus femoris (RF) and the medial gastrocnemius [12]. In contrast, a lower SWV in the medial gastrocnemius was observed in prepubertal boys than in men [13], but only after reaching long muscle lengths. This result highlighted the importance of considering the myotendinous length (i.e., joint angle) when considering the age effect.

The relationship between SWV and the myotendinous length is already well‐documented in adults [14]. This relationship is different between tendons and muscles [15]. Additionally, the presence of bi‐articular muscles complexifies the SWE evaluation. For instance, comparing the RF and the vastus lateralis (VL) implies taking into consideration both the hip and knee joints [4].

Considering the lack of knowledge about age‐related differences in the influence of knee and hip joint angles on myotendinous elastic properties, this study focused on determining the influence of the knee and hip angles on the SWV of VL, RF, and patellar tendon (PT) in prepubertal boys and men. It was expected to observe an increase of the SWV with increasing knee joint angle for VL, RF, and PT [14]. Additionally, we hypothesized that the hip joint would not influence the SWV values for VL and PT but that greater values would be observed for RF with a 0° hip joint angle. Finally, lower SWV values were expected in prepubertal boys than in men for both muscles and the PT.

## 2. Materials and Methods

### 2.1. Participants

Ten boys (9–11 years old) and 10 men (aged 20–25 years old) were recruited for this study (Table 1). The inclusion criteria required participants to have no reported lower body injuries in the 3 months before participation, to engage in at least 3 h of physical activity in a sport association per week, and for the boys to be prepubertal. To verify the prepubertal status, the age at peak height velocity (APHV) was calculated using the Mirwald formula [16]. A participant was considered prepubertal if the estimated age based on APHV exceeded their actual age. Additionally, the Tanner stage was estimated through self‐reported pubic hair development [17]. At stages one and two, the boys were considered prepubertal. Each participant was informed about the aim and the nature of the study. The study received approval from the local ethics committee (CERSTAPS: IRB00012476‐2023‐17‐02–229). The written informed consent was obtained before participation (legal representatives for boys). The sample size was determined a priori using G∗Power software (Version 3.1.9.6 available at https://www.psychologie.hhu.de/arbeitsgruppen/allgemeine-psychologie-und-arbeitspsychologie/gpower), with the following criteria: an effect size of 0.42, a power of 0.8, and a probability of error of 0.05, with the primary outcome being SWV. The effect size estimation was based on previous studies that observed large differences in tendon properties between children and adults [18, 19]. A sample size of 10 participants per group was indicated.

**Table 1:** Participant characteristics.

	Boys (*n* = 10)	Men (*n* = 10)
Age (years)	10.1 ± 0.9	21.0 ± 1.5
Height (cm)	141.6 ± 10.2	176.9 ± 7.6
Mass (kg)	33.2 ± 6.5	73.9 ± 7.6
APHV (years)	13.4 ± 0.4	—
Difference with APHV (years)	−3.3 ± 0.7	—
Tanner stage (pubic hair)	1‐2	—
Physical activity weekly (h)	5.4 ± 1.9	8.7 ± 3.8

*Note:* Values are means ± standard deviation. APHV: estimated age according to peak height velocity based on the Mirwald formula calculation with height, seated height, and mass.

### 2.2. Experimental Design

This study was a randomized trial. All volunteers came to the laboratory for one experimental session. All tests were conducted on the right side, ignoring the side dominance. Participants were asked to refrain from unusual activity the day before the session. The tests consisted of SWV measurements on VL, RF, and PT. The session started with the collection of anthropometric data and the control of the inclusion criteria. Then, the participants were positioned on an isokinetic ergometer to conduct the SWE data collection.

### 2.3. Ergometer

All measurements were realized passively on an isokinetic ergometer (Biodex 4 Quickset, Biodex Corporation, Shirley, NY, USA). The axis of rotation of the ergometer was aligned to the lateral femoral condyle. Participants were strapped to the pelvis to avoid any movement changing the position. The distal attachment arm of the ergometer was attached 1–2 cm above the lateral malleolus. The knee joint was tested at five knee angles going from extended to flexed knee joint: 30° (0°: full extension), 50°, 70°, 90°, and 110°. These five positions were tested with two different hip angles corresponding to 0° (full extension) and 80° (Figure 1). A total of 10 positions were tested (Figure 1), corresponding to: (1) Positions With an 80° Hip Joint Angle: H80‐K30, H80‐K50, H80‐K70, H80‐K90, and H80‐K110; and (2) Positions With a 0° Hip Joint Angle: H0‐K30, H0‐K50, H0‐K70, H0‐K90, and H0‐K110. All positions were tested in a randomized order generated with the website https://www.randomizer.org. All positions were realized in series, with the time to perform the measures corresponding to 2 minutes per position. The participants’ leg movements from one position to another were conducted manually by the investigator (angular velocity ≈ 30°.s^−1^).

Figure 1(a): Hip and knee joint positions. H: hip joint angle; K: knee joint angle; numbers indicated the joint angle. (b): Experimental setup with the probe positions (blue ellipses) represented for the rectus femoris, the vastus lateralis, and the patellar tendon. (c): Region of interest used for muscles and tendon investigation. The first line represents an acquisition for the vastus lateralis (VL), the second one is the rectus femoris (RF), and the third is the patellar tendon (PT). The B‐mode and the SWE are presented in the left and right columns, respectively. The areas considered for the SWV are represented by the white circle for the two muscles and by the white rectangle for the patellar tendon.

**Figure:**
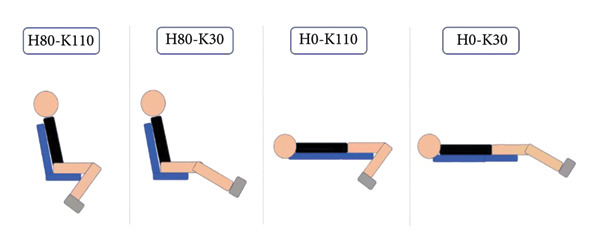
(a)

**Figure:**
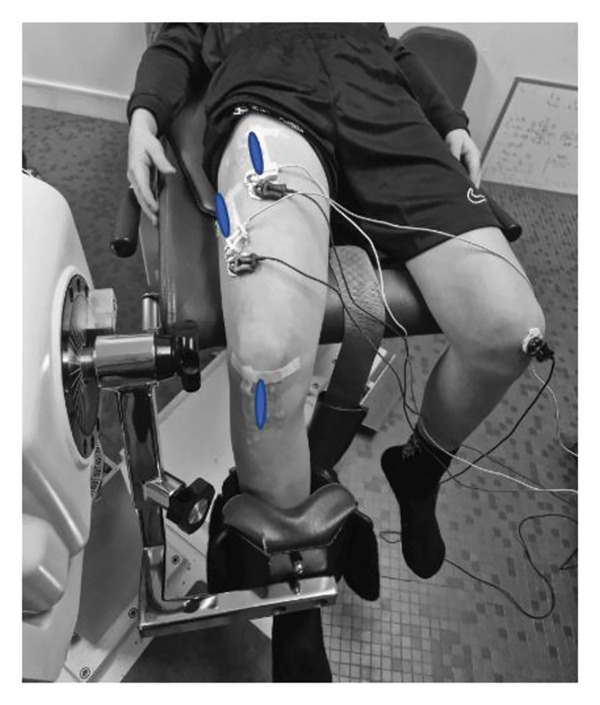
(b)

**Figure:**
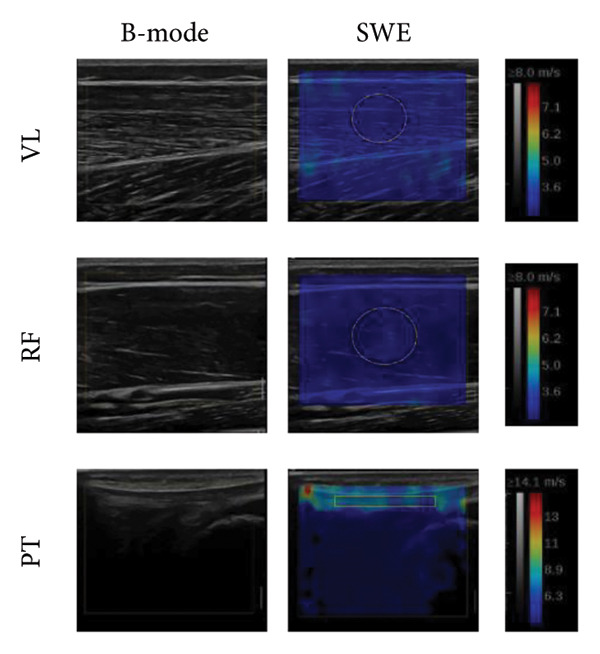
(c)

### 2.4. Elastography Measures

SWV was recorded using an Aixplorer ultrasound scanner (SuperSonic Imagine, model Aixplorer MACH30, Software Version 3.0.0 + SP2, Aix‐en‐Provence, France) with a linear probe (Linear 10‐2, Length: 45 mm, Width: 10 mm, SuperSonic Imagine, Aix‐en‐Provence, France). The B‐mode was used to determine the optimal probe position, respecting approximately probe position as follows: 25%–50% for RF, 50%–66% for VL (0%: greater trochanter; 100%: lateral femoral condyle) [20], and in contact with the distal edge of the patella for PT. The probe was positioned longitudinally to the muscles and tendon, in the central part of their belly for VL and RF. These locations were marked with straps on the skin to facilitate consistent measurements. Then, the SWE mode was used.

### 2.5. Data Collection

As recommended by previous studies, before starting the SWE acquisition, 10 preconditioning passive cycles were realized at 30°/s from 30° to 110° knee joint in a seated position [20, 21]. Two 8 s SWE videos were recorded successively by site and position. The SWE scale was 8.0 and 14.1 m.s^−1^, for muscle and tendon evaluations, respectively. These scales were chosen to reduce saturation risks resulting from high stiffness levels. To ensure the blinding about the image’s colorimetry, the opacity of the elastography map was calibrated at 0%. All measures were realized by a single investigator (BC) instructed and trained to minimize the probe pressure during SWE video acquisition and to maintain the probe perpendicularly to the aponeurosis of VL and RF.

Participants were asked to stay as relaxed as possible during all SWE measures. To control the absence of contraction, electromyographic activity of VL and RF was recorded using two pairs of silver chloride surface electrodes (ECG Electrodes, Kendall, CardinalHealth), with a 2 cm interelectrode distance. These electrodes were placed over the muscle (just near the ultrasound probe) after shaving, abrading, and cleaning the skin with alcohol. One reference electrode was attached to the left patella. The EMG signal was amplified with a bandwidth frequency ranging from 10 to 500 Hz (common mode rejection ratio = 110 dB, gain = 500). It was collected using a Biopac MP150 system and associated software (AcqKnowledge 4.2 for MP systems, Biopac System, Santa Barbara, CA, USA) at a 1000‐Hz sampling frequency. An activity outside of the interval of the mean ± 3 × standard deviation [22] observed after the session was an exclusion criteria. No participant was excluded.

### 2.6. Data Analysis

Six images separated by one second were extracted from each video. SWV values were averaged to obtain a representative value for each video. Then, values from the two videos were averaged for the final treatment. The SWV was obtained from the echograph software. For muscle investigation, the region of interest (ROI) was as large as possible (Figure 1), excluding aponeurosis [23]. For PT, the ROI position was the largest possible [24], excluding parts close to insertion on the bone [25] to avoid bone proximity artifacts [26].

### 2.7. Statistical Analyses

Statistical analyses were conducted using JASP (Version 0.17, JASP team 2020, University of Amsterdam). Shapiro–Wilk’s and Mauchly’s tests were used to test the normality and sphericity, respectively. A Greenhouse–Geisser correction was applied in case of nonsphericity. SWV was analyzed using a three‐way (age × knee angle × hip angle) analysis of variance (ANOVA) for PT and the two muscles separately. The “age” factor corresponded to men vs. boys. The “knee angle” corresponded to the five knee angulations (30°; 50°; 70°; 90°; and 110°). The “hip angle” corresponded to the two positions of the hip (0° and 80°). If significant main effects or interactions were revealed, post hoc tests with Bonferroni correction were conducted. Partial eta‐squares were reported as a measure of effect size and ranked as follows: 0.01 = small effect, 0.06 = moderate effect, and ≥ 0.14 = large effect [27]. Cohen’s d was also used for pairwise comparisons and ranked: < 0.5 = small, 0.5–1.2 = medium, and > 1.2 = large. To determine the reliability of the elastography measures, the intraclass correlation coefficient (ICC_(3.1)_) of Shrout & Fleiss [28] was used to compare the mean values obtained by the two videos.

For VL and RF, a linear relationship between the log value of the SWV and the knee angle for each individual was established. The slopes and y‐intercepts of the linear relationships were analyzed using a two‐way ANOVA (age × hip angle).

## 3. Results

The reliability of the measures assessed by ICC_(3.1)_ showed very good reliability with values > 0.9 (Table 2).

**Table 2:** Shear wave velocity intraclass correlation coefficient.

Position	Intraclass correlation coefficient (95% confidence interval)		
	VL	RF	PT
H0‐K30	0.968 (0.920; 0.988)	0.945 (0.862; 0.978)	0.987 (0.967; 0.995)
H0‐K50	0.980 (0.949; 0.992)	0.928 (0.817; 0.971)	0.987 (0.967; 0.995)
H0‐K70	0.944 (0.859; 0.978)	0.949 (0.871; 0.980)	0.993 (0.983; 0.997)
H0‐K90	0.967 (0.916; 0.987)	0.981 (0.952; 0.992)	0.970 (0.923; 0.988)
H0‐K110	0.976 (0.940; 0.991)	0.981 (0.952; 0.992)	0.966 (0.914; 0.987)
H80‐K30	0.961 (0.901; 0.984)	0.978 (0.943; 0.991)	0.988 (0.969; 0.995)
H80‐K50	0.929 (0.819; 0.972)	0.979 (0.947; 0.992)	0.993 (0.982; 0.997)
H80‐K70	0.963 (0.907; 0.985)	0.967 (0.917; 0.987)	0.993 (0.982; 0.997)
H80‐K90	0.963 (0.907; 0.985)	0.980 (0.948; 0.992)	0.927 (0.815; 0.971)
H80‐K110	0.986 (0.965; 0.994)	0.993 (0.981; 0.997)	0.933 (0.830; 0.973)

*Note:* The positions indicate the configurations of the hip and the knee joint angles. H0 and H80 correspond to a hip joint angle of 0° (lying position) and 80° (seated position), respectively. The knee joint flexion angle was indicated as follows: K30, K50, K70, K90, and K110, corresponding to knee joint flexion angles of 30, 50, 70, 90, and 110°, respectively.

### 3.1. SWV VL

**Figure 2:**
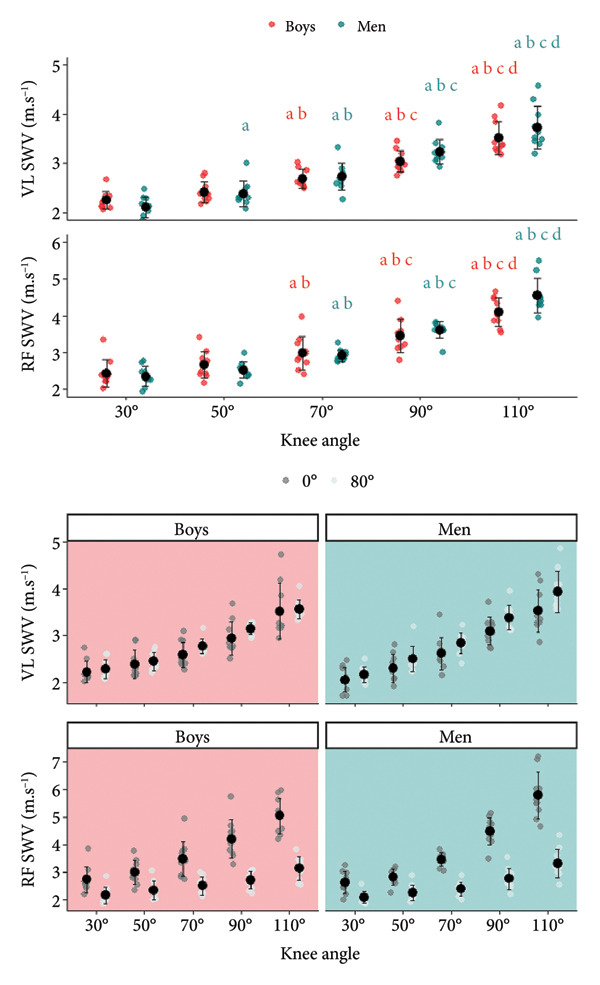
Vastus lateralis (VL) and rectus femoris (RF) shear wave velocity (SWV) dependence on knee and hip joint angles. Significant differences (*p* < 0.05) between knee angle positions are indicated as follows: 30° = a; 50° = b; 70° = c; 90° = d. No age effect was observed for all knee angles tested. Vastus lateralis (VL) and rectus femoris (RF) shear wave velocity (SWV) dependence on hip angle. A significant hip angle effect was observed, highlighting greater values in H0 (hip joint angle = 0°) compared to H80 (hip joint angle = 80°) for RF. For VL, greater values were observed in H80 compared to H0, whatever the age and knee angle.

A significant knee angle × age interaction was found for SWV VL (Figure 2; Table 3) (*p* = 0.008). Additionally, a significant hip angle effect was observed (*p* < 0.001). A significant difference was observed between all knee angles for men. For boys, a significant difference was also observed between all knee angles except between K30 and K50 (*p* = 0.676). Additionally, higher SWV VL values were observed in H80 as compared to H0 (*p* < 0.001; *d* = −0.998; mean difference with 95% CI [MD 95% CI]: −0.187 [−0.275; −0.099]). Boys and men did not present a difference in SWV VL values, whatever the knee angle considered.

**Table 3:** Results of the three‐way repeated‐measures ANOVA.

	Effect or interaction	*p*	*η* *p* ^2^
VL	Knee angle	**< 0.001** ^**∗∗∗**^	0.945
	Hip angle	**< 0.001** ^**∗∗∗**^	0.525
	Age	0.590	0.017
	Knee angle × hip angle	0.184	0.087
	Knee angle × age	**0.008** ^**∗∗**^	0.223
	Hip angle × age	0.143	0.115
	Knee angle × hip angle × age	0.352	0.057

RF	Knee angle	**< 0.001** ^**∗∗∗**^	0.925
	Hip angle	**< 0.001** ^**∗∗∗**^	0.901
	Age	0.606	0.015
	Knee angle × hip angle	**< 0.001** ^**∗∗∗**^	0.735
	Knee angle × age	**0.008** ^**∗∗**^	0.234
	Hip angle × age	0.414	0.037
	Knee angle × hip angle × age	0.211	0.084

PT	Knee angle	**< 0.001** ^**∗∗∗**^	0.926
	Hip angle	**< 0.001** ^**∗∗∗**^	0.700
	Age	**< 0.001** ^**∗∗∗**^	0.787
	Knee angle × hip angle	**0.003** ^**∗∗**^	0.195
	Knee angle × age	**< 0.001** ^**∗∗∗**^	0.289
	Hip angle × age	0.214	0.084
	Knee angle × hip angle × age	0.081	0.113

*Note:* ANOVA: analysis of variance; *η* *p* ^2^: partial eta‐squares. Significant effect or interaction was indicated as follows: ^∗∗^ *p* < 0.01; ^∗∗∗^ *p* < 0.001. The bold values represent the significant values.

Abbreviations: PT, patellar tendon; RF, rectus femoris; VL, vastus lateralis.

**Figure 3:**
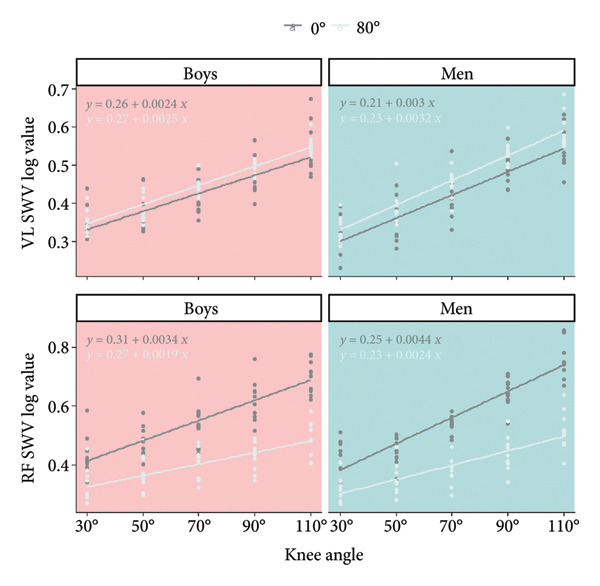
Vastus lateralis (VL) and rectus femoris (RF) shear wave velocity (SWV) log value dependence on knee and hip joint angles.

The slope and the *y*‐intercept of the log SWV for VL (Figure 3) were not influenced by the hip angle (*p* = 0.290; *η* *p* ^2^ = 0.062 and *p* = 0.077; *η* *p* ^2^ = 0.164). The slope of the log SWV for VL was greater for men than for boys (*p* = 0.003; *η* *p* ^2^ = 0.395; *d* = −0.767; MD 95% CI: −0.014 [−0.023; −0.005]). The *y*‐intercept was similar for the two age groups.

### 3.2. SWV RF

A significant knee angle × age interaction was found for SWV RF (*p* = 0.008). Additionally, a significant hip angle × knee angle interaction was observed (*p* < 0.001). A significant difference was observed between all knee angles for men and boys, except between K30 and K50 (*p* = 1.000). Additionally, higher SWV RF values were observed in H0 as compared to H80 for all knee angles. Boys and men did not present a difference in SWV RF values, whatever the knee angle considered.

The slope and the *y*‐intercept of the log SWV for RF were greater in H0 as compared to H80 (*p* < 0.001; *η* *p* ^2^ = 0.692; *d* = 1.423; mean difference with 95% CI: 0.035 [0.024; −0.047] and *p* = 0.002; *η* *p* ^2^ = 0.430; *d* = 0.824; MD 95% CI: 0.049 [0.021; 0.078]). Additionally, the slope of the log SWV for RF was greater for men than for boys (*p* = 0.026; *η* *p* ^2^ = 0.246; *d* = −0.542; MD 95% CI: −0.015 [−0.028; −0.002]). The *y*‐intercept was similar for the two age groups.

### 3.3. SWV Patellar Tendon

**Figure 4:**
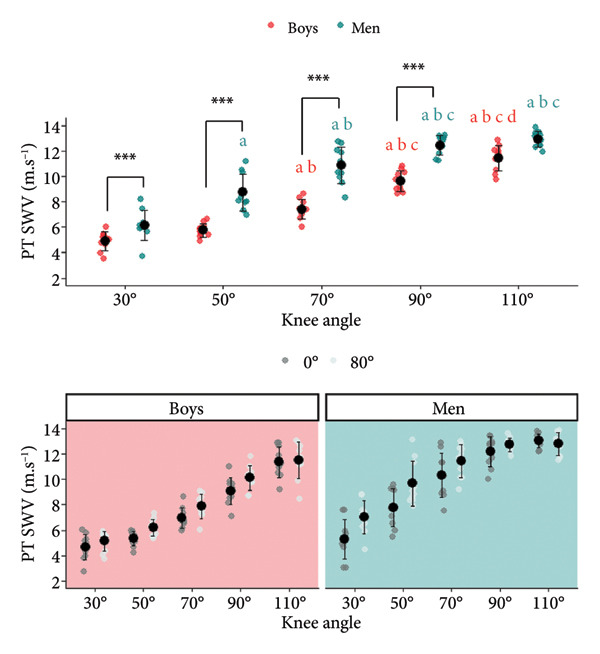
Tendon shear wave velocity (SWV) dependence on knee and hip joint angles. Significant differences (*p* < 0.05) between knee angle positions are indicated as follows: 30° = a; 50° = b; 70° = c; 90° = d. Significant differences between boys and men are represented as ^∗∗∗^ *p* < 0.001. Tendon shear wave velocity (SWV) dependence on hip angle. Significantly greater values were observed in H80 (hip joint angle = 80°) than H0 (hip joint angle = 0°) at K30 (knee joint flexion angle = 30°), K50 (knee joint flexion angle = 50°), and K70 (knee joint flexion angle = 70°).

A significant knee angle × age interaction was found for the SWV PT (Figure 4) (*p* < 0.001). Additionally, a significant hip angle × knee angle interaction was observed (*p* = 0.006). Significant differences were observed between all knee angles except between K90 and K110 (*p* = 1.000) for men. For boys, a significant difference was observed between all knee angles except between K30 and K50 (*p* = 0.999). Moreover, the SWV values for PT were greater in men as compared to boys at K30, K50, K70, and K90 (*p* < 0.001). Additionally, greater values were observed in H80 as compared to H0 at K30, K50, and K70.

## 4. Discussion

The aim of this study was to explore the influence of the knee and hip joint angles on the SWV, comparing men and prepubertal boys. For the three structures investigated (i.e., VL, RF, and PT), an increase of the SWV was observed as a consequence of the increase of the knee joint angle. This observation was congruent with the fact that the stiffness of the myotendinous tissues increases while it is lengthening. It was in accordance with previous studies using SWE in adults [14]. In addition to the influence of the knee joint angle, this study also explored the effect of the hip joint angles, comparing 0° and 80° angles. Our results confirmed that a 0° hip joint angle position would induce higher stiffness for RF as compared to 80°. Indeed, greater SWV values were found for RF with a 0° hip joint angle, along with a steeper increase in values, as supported by the greater slope observed in the log SWV RF/knee joint angle relationship. This finding was consistent with a previous study that compared 0° and 80° of hip angle [26]. These results for RF can be explained by the fact that it is a bi‐articular muscle crossing the knee and hip joints; as a consequence, a hip extension leads to greater lengthening compared to a flexed position. Additionally, the steeper increase in SWV observed with the 0° hip joint angle may be due to the quasiexponential nature of the relationship between SWV and knee joint angle at greater muscle lengths. In this context, 0° hip joint angle places the RF on the steeper portion of the SWV/knee angle curve.

The effects of hip angle for VL were more conflicting than those for RF. SWV values for VL were higher with an 80° hip joint angle as compared to 0°. Some studies found no effect of hip angle on monoarticular knee extensor muscles during passive stretching [29, 30], while one study [31] reported a higher VL stiffness with a 5° hip joint angle as compared to 90°. However, in the cited study [31], the participants were positioned lying on a table for both hip joint angles, whereas in this study, participants were positioned on an isokinetic ergometer, and changes in hip joint angle were associated with a modification of trunk inclination. Moreover, the raised‐leg position could lead to differences in the direction and magnitude of gravitational forces acting on the muscles and the associated structures. These methodological differences may have influenced the VL SWV. The interstudy difference could also be explained by the fact that some studies performed SWV measurements in proximal and distal muscle parts [29], while this study considered the central part of the muscle for the SWV measurements. Additionally, in some studies [14, 32], the SWV was collected during passive knee flexion, which involves continuous movement, whereas in this study, the position was maintained for about 2 minutes. It is reasonable to suggest that this difference could result in variations in the lengthening contribution of the various structures of the myotendinous complex. It has already been demonstrated that a 5‐min duration of static stretching can reduce gastrocnemius medialis SWV [33]. This hypothesis requires further investigation. It may be relevant to quantify the fascicle lengthening of VL while changing hip joint angle to assess the influence of hip joint angle on monoarticular knee extensor muscles. This may permit the identification of the contribution of individual muscles in the global myotendinous complex lengthening. Previous studies have already demonstrated that the hip configuration can influence VL architecture [34]. The use of 3D ultrasound may also be particularly interesting to improve our interpretations. However, considering the absence of difference between the two hip joint angles in the parameters of the log SWV VL/knee joint angle relationship, the influence of the hip joint angle on the SWV values for VL looks to be moderate.

Unexpectedly, no difference was observed between men and prepubertal boys in the SWV values for VL and RF, for all the configurations tested. Nevertheless, the results of this study were congruent with previous studies employing SWE [12] that demonstrated no age dependency for the RF elasticity. However, in the last cited study, a single position was tested for all participants and could potentially restrain the possibility of observing a difference. Indeed, in gastrocnemius medialis [13] significant difference between children (6–12 years old), adults (30–40 years old), and older individuals (55–66 years old) has been observed from 20° of dorsiflexion and beyond. The lower values were observed in children, followed by the adults, and then the older participants. A similar study was conducted with children from 2 to 12.6 years old, suggesting a greater passive stiffness of the lateral gastrocnemius for the older children, but without identifying a significant influence of age on the shear elastic modulus [11]. The interstudy difference could also be imputable to the consideration of different muscles and different myotendinous lengths.

While no main difference was observed between boys and men in the muscles’ SWV values, this study observed some age differences in the relationship between muscles’ SWV and knee joint angle (i.e., myotendinous complex length). Firstly, a significant difference in the SWV for VL between short positions (30° and 50°) was found for men, while no difference between the two knee joint angles was observed for prepubertal boys. Previous studies using SWE in men identified a significant increase in the shear elastic modulus around 50° of knee joint angle for VL and RF [14]. Additionally, the analysis of the slope of the log values of SWV for VL and RF revealed a lower slope for prepubertal boys. While the interpretation of this difference remains speculative, it may be due to a larger contribution of the tendon during myotendinous lengthening, potentially reducing intramuscular tension, which is supported by a less stiff tendon in boys. To substantiate this hypothesis, it would be necessary to identify architectural changes among the various tested positions to estimate the contributions of different structures. However, when considering the absence of difference between boys and men in the VL and RF SWV values, these differences in the relationship between the knee joint angle and the SWV values between the two age groups look subtle.

The SWV for PT increased as the knee angle increased in both boys and men, and the values observed in men were congruent with the literature [21]. For boys, these results constitute the first data with SWE and bring normative data. The hip joint angle also influenced the PT SWV. A greater tendon SWV was observed with an 80° hip joint angle as compared to 0°, from 30° to 70° knee joint flexion. This observation infirmed the hypothesis that hip joint angle may not influence PT SWV. This hypothesis was based on results from a previous study, which did not observe a difference in PT length and cross‐sectional area between supine and lying position [35]. It suggests that changes observed in this study in PT SWV between the two hip joint angles are only related to the passive force transmission from the muscles. As exposed for the results observed in VL, it is reasonable to suppose that hip joint angle influences the passive tension distribution between the knee extensor muscles and tendons. However, the interactions among various components of the myotendinous complex are intricate, rendering interpretations speculative. The interactions of the passive tension distribution among synergistic muscles within the same group are currently attributed to shared tendons [36]. Thus, it is plausible that PT plays an interplay role in the passive tension between knee extensor muscles. Moreover, connective tissues, such as an areolar connective tissue or a neurovascular connective tissue [37], constituting the interface between muscle bellies, could also induce an epimuscular force transmission [36]. Additionally, recent evidence demonstrated that knee joint angle affects the stiffness of the monoarticular muscle soleus in the lower leg [32]. One possible explanation involves myofascial transmission [38], but this hypothesis requires further investigation because there is no clear consensus about it for the knee extensor muscle group [29, 31]. All these phenomena may also contribute to the increase in VL SWV in a supine position. To clearly identify the influence of the hip joint angle, it is necessary to also consider the vastus medialis [31] and the other tendinous structures of the knee extensor complex.

A difference between boys and men was observed in the tendon properties, highlighting lower SWV in boys. A lower tendon stiffness for boys than for men has been observed with ultrasonography during active contraction [18, 19]. This lower tendon stiffness in children is currently attributed to a lower collagen fiber diameter [39], a reduced collagen cross‐linking [40], and a greater crimping [41]. In this study, this difference between boys and men was observed in a wide range of knee angle flexion (from 30° to 90° of knee joint angle flexion), while no significant difference was observed at a 110° knee flexion. The lack of difference between boys and men at 110° might suggest a similar passive tension in the tendon tissue in this configuration for the two age groups. Additionally, no saturation was indicated by the software; however, in another case, the absence of difference at a high knee flexion angle in tendon SWV could also be attributed to an underestimation of values due to the scale limit of the echograph when values approach 14.1 m.s^−1^. Therefore, to avoid this issue, it is recommended to use the highest available scale range to measure tendon tissue with the SWE.

In light of these results, it is plausible to propose some practical recommendations for using SWE in the pediatric population, also applicable in adults. Regarding muscle elastic properties, it may be pertinent to use a knee joint angle of at least 70° of flexion to ensure minimal tension. Indeed, the literature recommends using knee joint angles that allow for a detectable difference with knee extended positions [20]. Based on these results, the boys exhibited a significant increase with the extended (30°) knee joint position at a minimum of 70° of knee flexion. For a global analysis, including tendinous tissue, it is necessary to conduct measurements with conditions that avoid saturation issues. Therefore, it is advisable to remain below 90° of knee flexion to guarantee the absence of saturation. Consequently, a knee joint angle of around 70° could be appropriate. Regarding the hip joint angle, an influence was observed on both muscles and tendon tissue. This influence emphasizes the importance of considering the hip angle and adopting positions similar to those used in other studies in the research area to facilitate interstudy comparisons.

This study presents some limitations that lead to numerous perspectives. The participants were healthy and involved in a sports training context. Thus, similar measurements should be performed on a larger cohort, including trained and untrained participants, men and women, and pathological individuals, to establish more complete recommendations. Additionally, the SWE is a method that considers a local part of the tissue for the characterization of the elastic properties. However, some differences in the stiffness have already been observed, such as for RF [42]. Thus, the absence of difference between boys and men in the VL and RF SWV needs to be confirmed in the proximal and distal parts of these muscles. Finally, two methodological points require further investigation. First, the threshold used to confirm the absence of contraction was based on the mean EMG during a rest period. It may be relevant to confirm the use of this method by comparing with a threshold based on maximal voluntary contraction EMG [43]. Secondly, the maintained position used during the data collection may influence the myotendinous elastic properties by stretching the structures. This potential influence needs to be clearly identified.

## 5. Conclusion

This study compared the influence of the knee and hip joint angle on the stiffness of VL and RF muscles and PT between men and prepubertal boys. The results of this study suggested using a knee joint angle of around 70° of flexion to assess the knee extensor myotendinous properties with SWE in healthy individuals. Additionally, the results highlighted the influence of hip joint angle on the SWV for VL, RF, and PT. Thus, the hip joint angle has to be taken into consideration and should be standardized to facilitate the interstudy comparison.

For the two hip angles explored, no difference was observed between men and prepubertal boys for all the knee angles tested. The complementary analysis suggested a different relationship between the myotendinous length and muscles SWV, which is coherent with the fact that prepubertal boys present some differences, especially in the tendon properties that could affect the intramuscular tension during passive lengthening. However, it suggested that the muscle properties of the knee extensor muscle could be assessed in boys with similar conditions to men. In contrast, for the PT, lower SWV values were observed for boys than for men.

## Conflicts of Interest

The authors declare no conflicts of interest.

## Funding

No funding was received for this manuscript.

## Data Availability

The data that support the findings of this study are available from the corresponding author upon reasonable request.
